# Bis[2-(1*H*-benzimidazol-2-yl)benzoato]copper(II) dihydrate

**DOI:** 10.1107/S1600536810037633

**Published:** 2010-09-30

**Authors:** Jun Wang, Chuntao Dai, Dongmei Zhao

**Affiliations:** a Zhongshan Polytechnic, Zhongshan, Guangdong 528404, People’s Republic of China

## Abstract

In the title compound, [Cu(C_14_H_9_N_2_O_2_)_2_]·2H_2_O, the Cu(II) ion lies on a centre of symmetry and is four-coordinated by two N atoms and two O atoms from two 2-(1*H*-benzimidazol-2-yl)benzoate ligands in a square-planar environment. The benzimidazol and benzyl rings form a dihedral angle of 42.8 (5)°. The mol­ecule contains two H-bonded carboxyl O acceptors and two H-bonded N—H donors in the benzimidazol groups, which inter­act with two symmetry-related uncoordinated water mol­ecules so that neighboring mol­ecular units are linked by (O—H)_water_⋯O_carbox­yl_ hydrogen bonds with an *R* ^2^_4_(8) graph-set motif, generating a helical chain in the *a*-axis direction. These chains are, in turn, inter­connected by (N—H)_benzimidazol_⋯O_water_ hydrogen bonds, forming a three-dimensional supra­molecular network.

## Related literature

For the structural diversity and potential applications in functional materials of metal coordination polymers based on benzimidazole derivatives, see: Aminabhavi *et al.* (1986 ▶); Isele *et al.* (2005 ▶). For similar structures, see: Che *et al.* (2006 ▶); Fang *et al.* (2006 ▶); Liu *et al.* (2004 ▶); Li *et al.* (2010 ▶). For hydrogen-bond motifs, see: Bernstein *et al.* (1995 ▶).
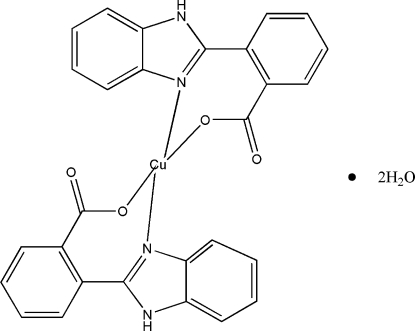


## Experimental

### 

#### Crystal data

[Cu(C_14_H_9_N_2_O_2_)_2_]·2H_2_O
*M* *_r_* = 574.04
Monoclinic, 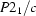

*a* = 11.6235 (2) Å
*b* = 7.6920 (2) Å
*c* = 16.1410 (3) Å
β = 115.735 (1)°
*V* = 1299.99 (5) Å^3^
*Z* = 2
Mo *K*α radiation
μ = 0.89 mm^−1^
*T* = 296 K
0.23 × 0.21 × 0.16 mm

#### Data collection

Bruker APEXII CCD area-detector diffractometer
Absorption correction: multi-scan (*SADABS*; Sheldrick, 2008*a* ▶) *T*_min_ = 0.821, *T*_max_ = 0.871
14480 measured reflections
2974 independent reflections
1854 reflections with *I* > 2σ(*I*)
*R*_int_ = 0.063

#### Refinement

*R*[*F* ^2^ > 2σ(*F* ^2^)] = 0.046
*wR*(*F* ^2^) = 0.110
*S* = 1.00
2974 reflections
178 parameters
H-atom parameters constrained
Δρ_max_ = 0.29 e Å^−3^
Δρ_min_ = −0.33 e Å^−3^

### 

Data collection: *APEX2* (Bruker, 2004 ▶); cell refinement: *SAINT* (Bruker, 2004 ▶); data reduction: *SAINT*; program(s) used to solve structure: *SHELXS97* (Sheldrick, 2008*b* ▶); program(s) used to refine structure: *SHELXL97* (Sheldrick, 2008*b* ▶); molecular graphics: *XP* in *SHELXTL* (Sheldrick, 2008*b* ▶); software used to prepare material for publication: *SHELXTL*.

## Supplementary Material

Crystal structure: contains datablocks I, global. DOI: 10.1107/S1600536810037633/bg2369sup1.cif

Structure factors: contains datablocks I. DOI: 10.1107/S1600536810037633/bg2369Isup2.hkl

Additional supplementary materials:  crystallographic information; 3D view; checkCIF report

## Figures and Tables

**Table 1:** Hydrogen-bond geometry (Å, °)

*D*—H⋯*A*	*D*—H	H⋯*A*	*D*⋯*A*	*D*—H⋯*A*
N2—H2*A*⋯O1*W*	0.86	1.86	2.716 (3)	173
O1*W*—H1*W*⋯O2^i^	0.84	1.89	2.720 (3)	167
O1*W*—H2*W*⋯O2^ii^	0.84	1.94	2.763 (3)	165

Symmetry codes: (i) 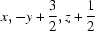; (ii) 
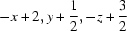
.

**Fig. 1.**
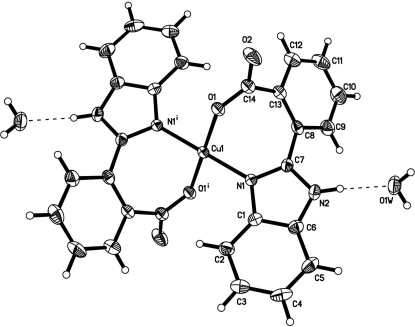
ORTEP represention of atom numbering diagram for (I), showing 30% probability displacement ellipsoids. Unlabelled atoms are related to the labelled atoms by the symmetry operator (i): 1-x, 1-y, 1-z.

**Fig. 2.**
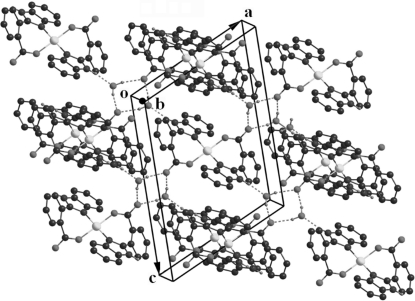
View of the three-dimensional structure of the title compound.

## supplementary crystallographic information

### Comment

Metal coordination polymers based on benzimidazole derivatives have raised intense interest for their structural diversity and their potential applications in functional materials (Aminabhavi *et al.*, 1986; Isele *et al.*, 2005). To date, numerous one-, two-, and three-dimensional coordination polymers have been synthesized by the choice of appropriate metal ions and versatile benzimidazole derivatives as ligands (Che *et al.*, 2006; Fang *et al.*, 2006; Liu *et al.*, 2004; Li *et al.*, 2010). Herein, the condensation of 1,2-diaminobenzene with 2-formylbenzoic acid in the presence of copper acetate lead to a new structure, Cu(C_14_H_9_N_2_O_2_)_2_.2H_2_O, the title compound herein reported .

As depicted in Fig. 1, the Cu^II^ ion lies on a centre of symmetry and is four-coordinated by two N atoms and two O atoms from two 2-(1*H*-Benzimidazol-2-yl)benzoate ligands in a square planar environment. The planar benzimidazol and benzyl rings form a dihedral angle of 42.8 (5)°. The molecule contains two H-bonded carboxyl O acceptors and two H-bonded N—H donors in the benzimidazol groups which interact with two symmetry-related lattice water molecules (symmetry code: 2 - *X*, 2 - *Y*, 2 - *Z*) in a way that neighboring molecular units are linked by (O—H)_water_···O_carbox_ hydrogen bonding with an *R*^2^_4_(8) graph set motif (Bernstein *et al.*, 1995) to generate a helical chain in the a-axis direction. These chains are in turn interconnected by (N—H)_benzimidazol_···O_water_ hydrogen bonds and extend to form a three-dimensional supramolecular network (table 1; Fig. 2)

### Experimental

The condensation reaction was done by reflux of 1,2-diaminobenzene (1.081 g; 10 mmol), 2-formylbenzoic acid (1.501 g; 10 mmol) and copper acetate (1.99 g; 10 mmol) in a hot 75% methanol/water (3:1; v/v) mixture (50 mL). Blue block crystals of the compound suitable for single-crystal X-ray diffraction analysis were obtained at room temperature by slow evaporation of the solvent (Yield 56% based on Cu).

### Refinement

All water H atoms were tentatively located in difference density Fourier maps and were refined with O–H distance restraints of 0.83 (1) Å and with U_iso_(H) = 1.5 U_eq_(O). In the last stage of refinement, they were treated as riding on their parent O atoms. All H atoms attached to C and N atoms were fixed geometrically and treated as riding with C—H = 0.93 Å and N—H = 0.86 Å, and U_iso_(H) = 1.2U_eq_(C) and U_iso_(H) = 1.5U_eq_(N).

### Crystal data

**Table:**

[Cu(C_14_H_9_N_2_O_2_)_2_]·2H_2_O	*F*(000) = 590
*M**_r_* = 574.04	*D*_x_ = 1.466 Mg m^−^^3^
Monoclinic, *P*2_1_/*c*	Mo *K*α radiation, λ = 0.71073 Å
Hall symbol: -P 2ybc	Cell parameters from 4800 reflections
*a* = 11.6235 (2) Å	θ = 1.4–28.0°
*b* = 7.6920 (2) Å	µ = 0.89 mm^−^^1^
*c* = 16.1410 (3) Å	*T* = 296 K
β = 115.735 (1)°	Block, blue
*V* = 1299.99 (5) Å^3^	0.23 × 0.21 × 0.16 mm
*Z* = 2	

### Data collection

**Table:**

Bruker APEXII CCD area-detector diffractometer	2974 independent reflections
Radiation source: fine-focus sealed tube	1854 reflections with *I* > 2σ(*I*)
graphite	*R*_int_ = 0.063
φ and ω scan	θ_max_ = 27.5°, θ_min_ = 1.9°
Absorption correction: multi-scan (*SADABS*; Sheldrick, 2008*a*)	*h* = −15→14
*T*_min_ = 0.821, *T*_max_ = 0.871	*k* = −9→9
14480 measured reflections	*l* = −20→20

### Refinement

**Table:**

Refinement on *F*^2^	Primary atom site location: structure-invariant direct methods
Least-squares matrix: full	Secondary atom site location: difference Fourier map
*R*[*F*^2^ > 2σ(*F*^2^)] = 0.046	Hydrogen site location: inferred from neighbouring sites
*wR*(*F*^2^) = 0.110	H-atom parameters constrained
*S* = 1.00	*w* = 1/[σ^2^(*F*_o_^2^) + (0.0379*P*)^2^ + 0.7646*P*] where *P* = (*F*_o_^2^ + 2*F*_c_^2^)/3
2974 reflections	(Δ/σ)_max_ = 0.001
178 parameters	Δρ_max_ = 0.29 e Å^−^^3^
0 restraints	Δρ_min_ = −0.33 e Å^−^^3^

### Special details

**Table:**

Geometry. All esds (except the esd in the dihedral angle between two l.s. planes) are estimated using the full covariance matrix. The cell esds are taken into account individually in the estimation of esds in distances, angles and torsion angles; correlations between esds in cell parameters are only used when they are defined by crystal symmetry. An approximate (isotropic) treatment of cell esds is used for estimating esds involving l.s. planes.
Refinement. Refinement of F^2^ against ALL reflections. The weighted R-factor wR and goodness of fit S are based on F^2^, conventional R-factors R are based on F, with F set to zero for negative F^2^. The threshold expression of F^2^ > 2sigma(F^2^) is used only for calculating R-factors(gt) etc. and is not relevant to the choice of reflections for refinement. R-factors based on F^2^ are statistically about twice as large as those based on F, and R- factors based on ALL data will be even larger.

### Fractional atomic coordinates and isotropic or equivalent isotropic displacement parameters (Å^2^)

**Table:**

	*x*	*y*	*z*	*U*_iso_*/*U*_eq_	
Cu1	0.5000	0.5000	0.5000	0.03258 (17)	
C1	0.5169 (3)	0.8319 (4)	0.6049 (2)	0.0354 (7)	
C2	0.4078 (3)	0.9168 (4)	0.5430 (2)	0.0439 (8)	
H2	0.3519	0.8648	0.4883	0.053*	
C3	0.3861 (4)	1.0813 (5)	0.5665 (3)	0.0511 (9)	
H3	0.3139	1.1414	0.5265	0.061*	
C4	0.4686 (4)	1.1600 (5)	0.6478 (3)	0.0582 (10)	
H4	0.4508	1.2721	0.6605	0.070*	
C5	0.5758 (4)	1.0775 (5)	0.7099 (3)	0.0535 (10)	
H5	0.6302	1.1301	0.7649	0.064*	
C6	0.5994 (3)	0.9111 (4)	0.6870 (2)	0.0400 (8)	
C7	0.6757 (3)	0.6520 (4)	0.6772 (2)	0.0332 (7)	
C8	0.7588 (3)	0.4981 (4)	0.7070 (2)	0.0358 (7)	
C9	0.8024 (3)	0.4489 (5)	0.7992 (2)	0.0504 (9)	
H9	0.7779	0.5126	0.8378	0.060*	
C10	0.8814 (4)	0.3068 (5)	0.8332 (3)	0.0632 (11)	
H10	0.9096	0.2750	0.8945	0.076*	
C11	0.9187 (3)	0.2119 (5)	0.7768 (3)	0.0604 (11)	
H11	0.9700	0.1141	0.7994	0.072*	
C12	0.8798 (3)	0.2620 (4)	0.6868 (2)	0.0502 (9)	
H12	0.9075	0.1995	0.6495	0.060*	
C13	0.7996 (3)	0.4051 (4)	0.6504 (2)	0.0367 (7)	
C14	0.7735 (3)	0.4580 (4)	0.5535 (2)	0.0386 (8)	
N1	0.5679 (2)	0.6681 (3)	0.60034 (16)	0.0327 (6)	
N2	0.6971 (2)	0.7946 (3)	0.73035 (17)	0.0413 (7)	
H2A	0.7614	0.8103	0.7828	0.050*	
O1	0.66231 (18)	0.4956 (3)	0.49508 (13)	0.0392 (5)	
O2	0.8661 (2)	0.4584 (4)	0.53508 (16)	0.0636 (8)	
O1W	0.8855 (2)	0.8417 (3)	0.90275 (15)	0.0624 (7)	
H1W	0.8680	0.8979	0.9406	0.094*	
H2W	0.9618	0.8657	0.9144	0.094*	

### Atomic displacement parameters (Å^2^)

**Table:**

	*U*^11^	*U*^22^	*U*^33^	*U*^12^	*U*^13^	*U*^23^
Cu1	0.0257 (3)	0.0338 (3)	0.0323 (3)	0.0030 (3)	0.0070 (2)	−0.0034 (3)
C1	0.0397 (19)	0.0320 (17)	0.0387 (18)	−0.0002 (14)	0.0210 (15)	−0.0016 (15)
C2	0.042 (2)	0.0385 (19)	0.049 (2)	0.0043 (16)	0.0178 (17)	0.0018 (17)
C3	0.054 (2)	0.041 (2)	0.066 (3)	0.0104 (19)	0.034 (2)	0.0083 (19)
C4	0.074 (3)	0.033 (2)	0.087 (3)	0.0080 (19)	0.053 (3)	−0.003 (2)
C5	0.064 (3)	0.042 (2)	0.061 (2)	−0.0088 (19)	0.032 (2)	−0.0177 (19)
C6	0.042 (2)	0.0360 (18)	0.046 (2)	−0.0016 (16)	0.0223 (17)	−0.0025 (16)
C7	0.0321 (17)	0.0363 (18)	0.0308 (16)	−0.0041 (14)	0.0132 (14)	−0.0034 (14)
C8	0.0265 (15)	0.0371 (16)	0.0371 (17)	−0.0048 (16)	0.0076 (13)	0.0013 (16)
C9	0.050 (2)	0.056 (2)	0.041 (2)	0.0050 (17)	0.0153 (17)	0.0057 (16)
C10	0.059 (3)	0.073 (3)	0.047 (2)	0.009 (2)	0.014 (2)	0.022 (2)
C11	0.051 (2)	0.059 (2)	0.066 (3)	0.023 (2)	0.021 (2)	0.029 (2)
C12	0.043 (2)	0.051 (2)	0.058 (2)	0.0089 (17)	0.0228 (18)	0.0113 (18)
C13	0.0288 (17)	0.0374 (18)	0.0396 (18)	0.0014 (14)	0.0109 (14)	0.0048 (15)
C14	0.0335 (19)	0.038 (2)	0.0420 (19)	0.0006 (14)	0.0142 (16)	0.0020 (14)
N1	0.0292 (14)	0.0314 (14)	0.0326 (14)	0.0024 (11)	0.0089 (11)	−0.0040 (11)
N2	0.0385 (16)	0.0449 (17)	0.0335 (14)	−0.0063 (13)	0.0090 (12)	−0.0094 (13)
O1	0.0296 (12)	0.0483 (13)	0.0362 (12)	0.0045 (11)	0.0111 (9)	0.0010 (11)
O2	0.0334 (14)	0.109 (2)	0.0530 (15)	0.0133 (14)	0.0232 (12)	0.0240 (14)
O1W	0.0453 (15)	0.0879 (19)	0.0496 (15)	−0.0167 (14)	0.0165 (12)	−0.0258 (14)

### Geometric parameters (Å, °)

**Table:**

Cu1—O1^i^	1.9227 (19)	C7—C8	1.470 (4)
Cu1—O1	1.9227 (19)	C8—C13	1.396 (4)
Cu1—N1	1.951 (2)	C8—C9	1.400 (4)
Cu1—N1^i^	1.951 (2)	C9—C10	1.379 (5)
C1—C2	1.390 (4)	C9—H9	0.9300
C1—C6	1.395 (4)	C10—C11	1.376 (5)
C1—N1	1.407 (4)	C10—H10	0.9300
C2—C3	1.374 (5)	C11—C12	1.376 (5)
C2—H2	0.9300	C11—H11	0.9300
C3—C4	1.384 (5)	C12—C13	1.396 (4)
C3—H3	0.9300	C12—H12	0.9300
C4—C5	1.370 (5)	C13—C14	1.513 (4)
C4—H4	0.9300	C14—O2	1.235 (4)
C5—C6	1.392 (5)	C14—O1	1.259 (3)
C5—H5	0.9300	N2—H2A	0.8600
C6—N2	1.376 (4)	O1W—H1W	0.8415
C7—N1	1.333 (3)	O1W—H2W	0.8432
C7—N2	1.347 (3)		
			
O1^i^—Cu1—O1	180.0	C13—C8—C7	124.1 (3)
O1^i^—Cu1—N1	90.25 (9)	C9—C8—C7	116.6 (3)
O1—Cu1—N1	89.75 (9)	C10—C9—C8	120.6 (3)
O1^i^—Cu1—N1^i^	89.75 (9)	C10—C9—H9	119.7
O1—Cu1—N1^i^	90.25 (9)	C8—C9—H9	119.7
N1—Cu1—N1^i^	180.0	C11—C10—C9	120.2 (3)
C2—C1—C6	120.9 (3)	C11—C10—H10	119.9
C2—C1—N1	131.1 (3)	C9—C10—H10	119.9
C6—C1—N1	108.0 (3)	C12—C11—C10	119.8 (3)
C3—C2—C1	117.0 (3)	C12—C11—H11	120.1
C3—C2—H2	121.5	C10—C11—H11	120.1
C1—C2—H2	121.5	C11—C12—C13	121.3 (3)
C2—C3—C4	122.0 (3)	C11—C12—H12	119.4
C2—C3—H3	119.0	C13—C12—H12	119.4
C4—C3—H3	119.0	C8—C13—C12	118.9 (3)
C5—C4—C3	121.8 (3)	C8—C13—C14	124.4 (3)
C5—C4—H4	119.1	C12—C13—C14	116.4 (3)
C3—C4—H4	119.1	O2—C14—O1	122.5 (3)
C4—C5—C6	116.9 (3)	O2—C14—C13	116.4 (3)
C4—C5—H5	121.5	O1—C14—C13	121.1 (3)
C6—C5—H5	121.5	C7—N1—C1	106.4 (2)
N2—C6—C5	132.7 (3)	C7—N1—Cu1	126.1 (2)
N2—C6—C1	105.9 (3)	C1—N1—Cu1	127.5 (2)
C5—C6—C1	121.4 (3)	C7—N2—C6	108.8 (3)
N1—C7—N2	110.9 (3)	C7—N2—H2A	125.6
N1—C7—C8	126.7 (3)	C6—N2—H2A	125.6
N2—C7—C8	122.3 (3)	C14—O1—Cu1	132.8 (2)
C13—C8—C9	119.2 (3)	H1W—O1W—H2W	106.7

Symmetry codes: (i) −*x*+1, −*y*+1, −*z*+1.

### Hydrogen-bond geometry (Å, °)

**Table:**

*D*—H···*A*	*D*—H	H···*A*	*D*···*A*	*D*—H···*A*
N2—H2A···O1W	0.86	1.86	2.716 (3)	173
O1W—H1W···O2^ii^	0.84	1.89	2.720 (3)	167
O1W—H2W···O2^iii^	0.84	1.94	2.763 (3)	165

Symmetry codes: (ii) *x*, −*y*+3/2, *z*+1/2; (iii) −*x*+2, *y*+1/2, −*z*+3/2.
